# Supplementary material for: Partial or complete blocking of gD binding to HVEM affects primary and latent HSV-1 infection as well as eye disease in HSV-1 infected mice
Source: PLoS Pathog. 2026 May 26;22(5):e1014258. doi: 10.1371/journal.ppat.1014258 (PMC13221150; doi:10.1371/journal.ppat.1014258)
Supplement: Attachment — Submitted filename: Response (04-28-2026)PP.doc [file ppat.1014258.s002.doc]

**RE**: PPATHOGENS-D-26-00687 (Partial or complete blocking of gD binding to HVEM affects primary and latent HSV-1infection as well as eye disease in HSV-1 infected mice)

Dear Drs. Shukla, Damania, Bhaduri-McIntosh, and Malim:

Thank you very much for considering our revised manuscript. Below are our detailed responses to each reviewer's comments, indicating the changes we have made. Thank you for the opportunity to revise and resubmit our manuscript.

**Reviewer #1:**

Herpes simplex virus type 1 (HSV-1) glycoprotein D (gD) plays a major player of virus entry, in large part because it interacts with heparan, HVEM. Previous studies revealed that amino acid 27-29 completely blocked gD binding to HVEM. This was the rational for mutating amino acid 27 and a mutant virus aa 27-29. These mutants were in the McKrae strain of HSV-1, which is a highly virulent strain of HSV-1. These mutant viruses were used to compare viral replication, corneal disease, latency, reactivation form latency, and T cell exhaustion. This study revealed that the ability of gD to interact with HVEM is crucial for viral replication and reactivation from latency. In general, this is an interesting study because interfering gD-HVEM can be used to develop a vaccine or small molecules that blocks the binding of HVEM to gD. With that said, there are a few minor issues that need to be addressed.

*RESPONSE*: Please see our responses to the reviewers' comments under Reviewers 1 & 2 below.

**Reviewer #2:**

Summary: Using a well characterized mouse model of HSV1 corneal infection, latency, and reactivation, Arya and coworkers performed a series of studies to investigate with greater precision the binding of virus-encoded glycoprotein D (gD) to host cell receptor HVEM based on an observation that gD amino acids Q27 – T29 play a central role in blocking gD binding to HVEM. Thus, two mutant viruses on a McKrae virus strain background were constructed, one with a single gD amino acid mutation (v27) and the other with three gD mutations (v27 – 29). The v27 mutant virus, the v27 – 29 mutant virus, and WT McKrae virus constructed to express GFP (GFP-McKrae) were compared for a number of experimental endpoints that included amounts of infectious virus in tear films, corneal scarring, angiogenesis, copies of LAT per ug of trigeminal ganglion RNA, copies of GFP and gD per ug of trigeminal ganglion RNA, CD8alpha and PD-1 expression, and reactivation of virus from explanted trigeminal ganglia. Importantly, these experimental endpoints were determined in mice with or without corneal scarification prior to HSV1 infection. Overall, v27 and v27 – 29-infected mice showed reduced amounts for all experimental endpoints examined when compared with mice infected with WT GFP-McKrae virus. Importantly, corneal scarification did indeed impact findings with mice with scarified corneas showing increased amounts for most experimental endpoints examined when compared with mice not subjected to corneal scarification. The authors conclude that disrupting gD-HVEM reduces virus virulence and limits latency, reactivation, and T cell exhaustion. The Q27 – T29 region of gD is therefore a critical determinant of infection.

Review: This is a well written manuscript that summarizes the findings of a series of relatively straightforward studies that provide convincing outcomes. These studies represent a nice extension of previous work by this research group that provides new and important information on how gD-HVEM interaction affects acute HSV1 replication at the corneal surface (especially if scarified or not scarified) as well as HSV1 latency and reactivation. Attention to a few issues might improve the manuscript and make it more reader friendly, however.

**Issue 1**. The authors surprisingly use GFP expression as an experimental endpoint to compare v27 v27 – 29, and WT McKrae viruses (Fig 4). More information is therefore needed about the precise construction of WT GFP-McKrae in the Materials and Methods section even though a previous paper from the literature is cited. This is needed because the GFP molecule is not naturally encoded by HSV1 and this molecule expression could be considered highly artificial. Where in the virus genome is the GFP gene placed? Is it expressed as an immediate-early, early, or late gene product? Would use of a virus-encoded immediate early or early gene product serve better than GFP expression from an artificially recombinant virus genome?
*RESPONSE*: A detailed methodology section was added to the text, explaining the construction of the v27 and v27-29 viruses from lines# 329 to 343.

**Issue 2**. It is noteworthy that a number of figures show outlier values (see Fig 4 and 5). Would the authors please comment on these outlier values and the possible reason(s) for their appearance?

*RESPONSE*: In HSV-1 infection dynamics, these outliers in these experiments are typically due to initial replication in the eyes, genetic background, immune cell status, and latency establishment in the TG. However, Figs. 4 and 5 may also indicate viral variants or subpopulations with a more pronounced phenotype, particularly strains with greater receptor usage flexibility. Some investigators may exclude these outliers, but we believe their exclusion is probably not proper.

**Issue 3**. Line 37 of the Abstract states “… that disrupting gD-HVEM inhibition reduces viral virulence …” Is use of the word “inhibition” correct in this sentence?

*RESPONSE*: The statement has been revised by replacing “inhibition” with “binding.”

**Reviewer #3:**

In this study, the authors used HSV-1 McKrae strain with mutations in gD protein, which prevented gD binding efficacy to HVEM. As a result, the authors showed reduced viral virulence, latency, reactivation and T cell exhaustion. The study used mouse model of corneal HSV-1 infection with or without corneal scratching. This is an interesting study, which highlights the outcome of gD-HVEM interaction during corneal HSV-1 infection. There are few minor concerns that authors should address to enhance the impact of their findings-

**Minor concern 1**. The authors should provide HSV-1 strain name in Figure 1 legend.
*RESPONSE*: The individual virus names have been inserted into the figure legend on lines #548- #550.

**Minor concern 2**. What was the rationale for choosing D28 post-infection for the clinical scoring of disease?

*RESPONSE*: Day 28 is often used as an endpoint in ocular HSV-1 mouse models because it captures the later phase of infection, when acute viral replication has already declined and the main readouts shift toward residual corneal disease, inflammation, and recovery- or latency-related outcomes. In one mouse study, eye disease was monitored through day 28, and the trigeminal ganglia were also examined on day 28 for latent infection and reactivation-related analyses.

**Minor concern 3**.HSV-1 McKrae is a highly neurovirulent strain and after corneal scratching, it may induce encephalitis. Did the authors note encephalitis in the corneal scratching group?

*RESPONSE*: Corneal scarification increased the virus replication in the eyes (Fig. 1B). Encephalitis is plausible in those animals, but was not measured here.

**Minor concern 4**. What could be the possible reason of reduced PD-1 level on T cells in the TG of mice receiving mutant virus?

*RESPONSE*: This has been explained in the manuscript text from lines #281-286.

Thank you very much for allowing us to revise and resubmit our manuscript. We trust that we have satisfactorily addressed these helpful reviewers’ comments and have now made all requested changes. New text in the body of the manuscript is printed in “Blue Arial” rather than “Black Arial”. We hope that the manuscript will now be deemed acceptable for publication in PLOS Pathogens.

Best Regards,

Homayon Ghiasi
